# Dissecting the Molecular Mechanisms of Neurodegenerative Diseases through Network Biology

**DOI:** 10.3389/fnagi.2017.00166

**Published:** 2017-05-29

**Authors:** Jose A. Santiago, Virginie Bottero, Judith A. Potashkin

**Affiliations:** Department of Cellular and Molecular Pharmacology, The Chicago Medical School, Rosalind Franklin University of Medicine and ScienceNorth Chicago, IL, United States

**Keywords:** Alzheimer’s disease, Parkinson’s disease, Huntington’s disease, network biology, molecular mechanisms

## Abstract

Neurodegenerative diseases are rarely caused by a mutation in a single gene but rather influenced by a combination of genetic, epigenetic and environmental factors. Emerging high-throughput technologies such as RNA sequencing have been instrumental in deciphering the molecular landscape of neurodegenerative diseases, however, the interpretation of such large amounts of data remains a challenge. Network biology has become a powerful platform to integrate multiple omics data to comprehensively explore the molecular networks in the context of health and disease. In this review article, we highlight recent advances in network biology approaches with an emphasis in brain-networks that have provided insights into the molecular mechanisms leading to the most prevalent neurodegenerative diseases including Alzheimer’s (AD), Parkinson’s (PD) and Huntington’s diseases (HD). We discuss how integrative approaches using multi-omics data from different tissues have been valuable for identifying biomarkers and therapeutic targets. In addition, we discuss the challenges the field of network medicine faces toward the translation of network-based findings into clinically actionable tools for personalized medicine applications.

## Introduction

**Figure 1:**
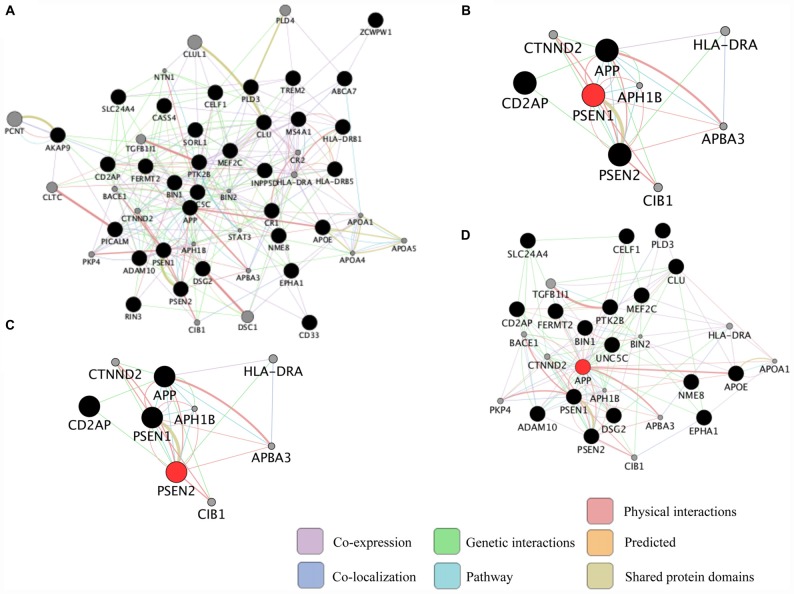
Representation of common biological networks. **(A)** Example of a network of interactions among genetic risk factors for Alzheimer’s disease (AD; black circles) and other related genes (gray circles). The color of the lines represents the type of interaction and the thickness is proportional to the strength of the association. **(B–D)** Presenilin 1 (PSEN1), PSEN2 and amyloid precursor protein (APP; red circles) are highly connected genes (hub genes) identified in the network. Hub genes usually play a central role in the disease. These networks were retrieved by GeneMANIA application in Cytoscape 3.1.1 as of September 2016 using the default settings to include the top 20 related genes and automatic weighting.

**Figure 2:**
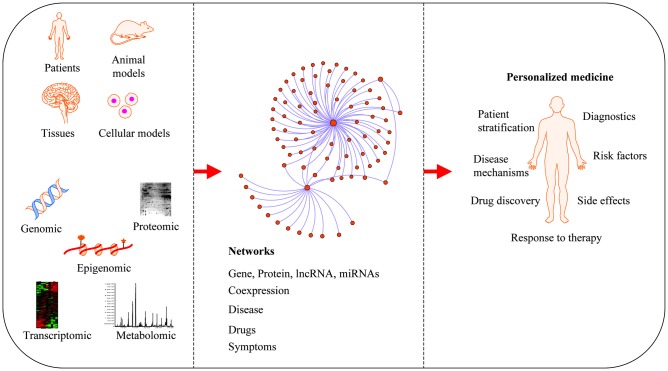
Applications of network medicine. Biological networks can be constructed from a wide range of different omic approaches including genomic, transcriptomic, epigenomic, metabolomic and proteomic datasets. In protein-protein interaction (PPI) networks, proteins are the nodes and their interactions are the edges. Network-based approaches have advanced the field of personalized medicine by providing novel mechanisms of disease, diagnostics and therapeutic targets.

Neurodegenerative diseases are usually sporadic in nature and commonly influenced by a wide range of genetic, epigenetic and environmental factors. With the advent of new high-throughput technologies such as RNA sequencing, it has become essential to develop methods beyond the classical pathway analysis to systematically interpret large amounts of data in the context of health and disease. Despite the progress of high-throughput genomic studies the precise pathogenic mechanisms leading to the most prevalent neurodegenerative diseases remain elusive. To this end, the applications of network biology have been successful to provide biological insight and to decipher the molecular underpinnings of neurodegenerative diseases. Network biology is based on the premise that complex diseases, like neurodegenerative diseases, are frequently caused by alterations in many genes comprising multiple biological pathways. A network consists of nodes and edges that may represent genes, proteins, miRNAs, noncoding RNAs, drugs, or diseases connected through a wide range of interactions including, but not limited to physical, genetic, co-expression and colocalization. An example of network analysis of Alzheimer’s disease (AD) that identifies central hubs is shown in Figure 1. Integration of multi-omic information coupled with network-based approaches is becoming an essential step towards the advancement of personalized medicine (Figure 2). Some of the frequently used terms in network biology approaches are defined in Table 1.

**Table 1:** Frequently used terms in network biology.

Term	Definition
Epigenetic	Epigenetic studies genetic effects not encoded in the DNA sequence of an organism.
Gene ontology (GO)	Gene ontology is a major bioinformatics initiative to unify the representation of gene and gene product attributes across all species.
Genome wide association study (GWAS)	A genome wide association study is an examination of the entire genome that is useful to identify genetic variants (SNPs) associated with a trait of interest.
Module	Module is defined as a group of physically or functionally linked molecules that work together to achieve a relatively distinct function. Modules are also called groups, clusters or communities. Examples of modules are co-regulation, co-expression, membership of a protein complex, of a metabolic or signaling pathway.
Network analysis	Network analysis is a method to systematically analyze a group of interconnected components. Nodes and edges are the basic components of a network. Nodes represent units in the network and edges represent the interactions between the units. Hubs are nodes with high connectivity.
Network medicine	Network medicine is an emerging field of network biology that applies the principles that govern cellular and molecular networks in the context of health and disease.
-omes	-omes are large scale networks. Interactome refers to the entire set of interactions in a particular cell. These interactions could represent, for example, protein-protein interactions (PPI) or interactions between messenger RNA molecules, also known as the transcriptome.
Single nucleotide polymorphism (SNP)	A single nucleotide polymorphism is a variation in a single nucleotide that occurs at a specific position in the genome. They are the most common type of genetic variation among people.
Weighted gene co-expression network analysis (WGCNA)	Weighted gene co-expression network analysis, also known as weighted correlation network analysis (WCNA), represents a systems biologic method for analyzing microarray data, gene information data, and microarray sample traits (e.g., case control status or clinical outcomes). WGCNA facilitates a network-based gene screening method that can be used to identify candidate biomarkers or therapeutic targets.

Seminal work in network biology including the construction of the human disease network (Goh et al., 2007), the human functional linkage network (Linghu et al., 2009), the discovery of causal genes of obesity (Chen et al., 2008), and clinical biomarkers for cancer (Taylor et al., 2009), prompted efforts to study many different diseases using network-based approaches. In the last few years, there has been a steady growth in studies exploiting the concepts of network biology to understand neurodevelopmental and neurodegenerative diseases (Santiago and Potashkin, 2014a). For example, network approaches have successfully identified putative diagnostic biomarkers for Parkinson’s disease (PD; Santiago and Potashkin, 2013a, 2015; Santiago et al., 2014, 2016), and progressive supranuclear palsy (Santiago and Potashkin, 2014b) reviewed in Santiago and Potashkin (2013b, 2014a,c). In addition, network-based approaches have provided insights into the molecular mechanisms underlying co-morbid diseases associated with PD including diabetes (Santiago and Potashkin, 2013a) and cancer (Ibáñez et al., 2014). In this review article, we highlight the most recent advances in network biology applications to understand the most common neurodegenerative diseases with an emphasis on brain specific networks.

## Network-Based Approaches Identifies Pathways Specific to Alzheimer’S Disease (AD)

AD is the most prevalent neurodegenerative disease, responsible for the majority of the cases of dementia, affecting more than 44 million people worldwide with an estimated global cost of more than 600 billion dollars^1^. Although the exact mechanism of disease remains unclear, a complex combination of genetic, epigenetic, lifestyle, environmental factors and aging are believed to be responsible for most of the cases. Pathological features of AD include the accumulation of amyloid beta (Aβ) plaques and protein tau in neurofibrillary tangles (NFT). While most of the AD cases are late onset (LOAD) and sporadic, some genetic mutations in the amyloid precursor protein (*APP*), presenilin 1 (*PSEN1*) and presenilin 2 (*PSEN2*) are documented to cause early onset AD, which accounts for approximately 2% of the cases with symptoms appearing before the age of 65 (Goate et al., 1991; Levy-Lahad et al., 1995; Janssen et al., 2003). The apoliporotein E-ε4 (APOEε4) is the only genetic factor identified in more than 60% of the sporadic AD cases, however, it has also been found in healthy individuals thus suggesting that other genetic factors may be responsible for the disease (Coon et al., 2007). To date, emerging high-throughput genomic technologies have reported more than 2900 genetic variations associated with AD^2^.

Although these studies have been valuable to understand the genetic diversity associated with AD, the multi-factorial mechanisms leading to the disease are unclear. Network-based approaches have been successful to systematically interpret these results and to gain insight into the mechanisms of disease. In particular, integrative approaches combining multi-omic data in networks have been employed to identify susceptibility genes and pathways in AD. For example, combinatorial network analysis of proteomic and transcriptomic data revealed subnetworks enriched in pathways associated with the pathogenesis of AD including the downregulation of genes associated with the *MAPK/ERK* pathway and the upregulation of genes associated to the clathrin-mediated receptor endocytosis pathway (Hallock and Thomas, 2012; Table 2). In this regard, disruption of the clathrin-mediated receptor pathway can lead to increased levels of APP thereby contributing to disease progression (Schneider et al., 2008; Hallock and Thomas, 2012). Integrative approaches have led to the identification of potential additional genetic risk factors and biomarkers for AD. For instance, integration of genome wide association studies (GWAS), linkage analysis and expression profiling in a protein-protein interaction (PPI) network yielded a 108 potential risk factors for AD including EGFR, ACTB, CDC2, IRAK1, APOE, ABCA1 and AMPH. Among these genes, EGFR, APOE and ACTB were found to overlap with proteomic data from cerebrospinal fluid of AD patients (Talwar et al., 2014) thus providing potential biomarker candidates. Collectively, these studies reinforce the power of integrative network approaches to identify pathways, genetic risk factors and biomarkers for AD.

**Table 2:** Brain network-based analysis of the most common neurodegenerative diseases.

Disease	Networks identified	Reference
AD	*MAPK/ERK* and clathrin-mediated receptor endocytosis	Hallock and Thomas (2012)
	Immune system and microglia	Zhang et al. (2013)
	Astrocyte-specific and microglia-enriched modules	Miller et al. (2013)
	Myelination and innate immune response	Humphries et al. (2015)
	Network modules of AD progression	Kikuchi et al. (2013)
	Co-expression modules based on *APOEɛ4* stratification	Jiang et al. (2016)
	Downregulated network of genes corresponding to metastable proteins prone to aggregation	Ciryam et al. (2016)
	Hypomethylation patterns in a myelination network	Humphries et al. (2015)
PD	Stress response and neuron survival/degeneration mechanisms	Corradini et al. (2014)
	Key protein targets including p62, GABARAP, GBRL1 and GBRL2 that modulated 1-methyl-4-phenylpyridinium (MPP^+^) toxicity	Keane et al. (2015)
	Alvespimycin neuroprotective agent for PD	Gao et al. (2014)
	RGS2 as a key regulator of LRRK2 function	Dusonchet et al. (2014)
	Downregulation of RNA and protein expression of a network of transcription factors *FOXA1, NR3C1, HNF4A* and *FOSL2*	Fernández-Santiago et al. (2015)
HD	Modules associated with *Htt* CAG length and toxicity	Langfelder et al. (2016)
	Metalloprotein, stress response, angiogenesis, mitochondrion, glycolysis, intracellular protein transport, proteasome, synaptic vesicle	Neueder and Bates (2014)
	Protein modification, vesicles transport, cell signaling and synaptic transmission	Mina et al. (2016)
	Astrocyte module associated with TGFβ -FOXO3 signaling, stress and sleep phenotype	Scarpa et al. (2016)
Aging, neurodegeneration	DNA repair, RNA metabolism, and glucose metabolism shared in AD and PD	Calderone et al. (2016)
	242 genes enriched in pathways related to neuron differentiation, apoptosis, gap junction trafficking, and cellular metabolic processes in AD and HD	Narayanan et al. (2014)
	Inflammation, mitochondrial dysfunction, and metal ion homeostasis in aging and PD	Glaab and Schneider (2015)
	Chaperome critical to maintain protein homeostasis in aging and neurodegeneration	Brehme et al. (2014)

Weighted gene coexpression networks analysis (WGCNA) are increasingly being used to find highly co-expressed gene modules associated with a particular biological pathway or a clinical trait of interest (Langfelder and Horvath, 2008). For example, construction of gene co-expression networks from 1647 postmortem brain tissues from LOAD patients highlighted immune and microglia enriched modules, containing a key regulator of the immune system, known as TYROBP (Zhang et al., 2013). Likewise, WGCNA analysis uncovered astrocyte-specific and microglia-enriched modules in vulnerable brain regions that associated with early tau accumulation (Miller et al., 2013). Implementation of WGCNA in RNA-sequencing data using brain samples obtained from the temporal lobe of subjects with dementia with Lewy body (DLB), LOAD and cognitively normal patients, identified network modules specific to each disease. For example, two network modules enriched in myelination and innate immune response correlated with LOAD whereas network modules associated with synaptic transmission and the generation of precursor metabolites correlated with DLB and LOAD (Humphries et al., 2015). Further, genes previously implicated in LOAD including *FRMD4B* and *ST18* (Miller et al., 2008; Zhang et al., 2013) were prominent hubs within the myelination network (Humphries et al., 2015). Together, these findings suggested the involvement of microglia and myelination in the pathogenesis of AD and established differences in biological pathways between LOAD and DLB. Besides innate immunity pathways, network analysis of transcriptomic data from the brain hippocampus of normal aged and AD subjects identified key transcriptional regulators related to insulin (*INS1, INS2*) and brain derived neurotrophic factor (*BDNF*) interacting with the retinoic acid receptor related orphan receptor (*RORA*, Acquaah-Mensah et al., 2015) previously implicated in autoimmunity and diabetes (Solt and Burris, 2012).

With the growing interest in personalized medicine, it has become essential to develop tools to stratify patients according to symptoms, prognosis, and disease stage. This is highly important due to the fact that some subgroups of patients within a specific disease may experience a faster disease progression or respond to therapy differently. There are several documented examples on how networks could accelerate individualized treatment. For instance, analysis of protein interaction networks identified unstable network modules in different brain regions, in particular, in the entorhinal cortex of AD patients. Specifically, several protein interactions were present or absent at different Braak stages thus providing network modules characteristic of disease progression in AD (Kikuchi et al., 2013). Interestingly, the network modules with the largest number of disappearing protein interactions at late stage were associated with the histone acetyltransferase and the proteasome complexes. These modules were interacting *via* UCHL5 thereby indicating the perturbation of the ubiquitin-proteosome system in AD. Likewise, network analysis of six relevant brain regions affected in AD uncovered 136 hub genes of which 72 correlated with the Mini Mental State Examination (MMSE) and NFT scores, both widely utilized indicators of disease severity in AD (Liang et al., 2012). Among these genes, there were important transcription factors and kinases associated with AD including *LEF1, SOX9, YY1, TCF3, TFDP1, CDK5, CSK* and *MAP3K3*. Among these genes, overactivation of *CDK5* is a major trigger of tau hyperphosphorylation and NFT formation in AD suggesting it may be a target for therapeutic intervention (Wilkaniec et al., 2016).

Since some genetic risk factors have a stronger influence in the disease than others, patient categorization and stratification according to the genetic basis would be advantageous in personalized medicine. In the context of AD, *APOEɛ4* is the strongest risk factor for LOAD accounting for more than 50% of the cases. *APOEɛ4* carriers display different clinical and pathological features than those of non-carriers. For example, *APOEɛ4* carriers perform worse on memory tasks (Marra et al., 2004) and have a higher amyloid beta deposition than non-carriers (Kandimalla et al., 2011; Jack et al., 2015). Moreover, *APOEɛ4* carriers respond to treatment differently than non-carriers. For instance, a neuroprotective agent improved MMSE scores in *APOEɛ4* carriers but not in non-carriers (Richard et al., 1997). WGCNA on a transcriptomic dataset from human cerebral cortex of LOAD identified distinct co-expression modules based on *APOEɛ4* stratification (Jiang et al., 2016). Co-expression modules of *APOEɛ4* carriers were enriched in hereditary disorders, neurological diseases, and nervous system development and function whereas modules of non-carriers were enriched in immunological and cardiovascular diseases thus suggesting that different biological processes could play a role in LOAD with different APOEε4 status (Jiang et al., 2016).

## Network-Based Approaches in Parkinson’S Disease (PD)

PD is the second most prevalent neurodegenerative disease after AD, affecting more than 10 million people worldwide. Pathological features include the accumulation of aggregated alpha synuclein (SNCA) in intraneuronal cytoplasmic inclusion known as Lewy bodies and the progressive loss of dopaminergic neurons in the substantia nigra pars compacta. Dopamine restorative drugs and deep brain stimulation are current therapies to treat patients, however, these treatments only alleviate motor symptoms but do not impact disease progression. Mutations in the *LRRK2, PARK2, PARK7, PINK1* and *SNCA* genes are known to cause familial PD. Most of the PD cases, however, are sporadic resulting from a complex interplay between genetics and environmental factors. In fact, some of the same genetic variants including *SNCA* and *LRRK2* implicated in familial PD have been also associated with sporadic PD (Satake et al., 2009; Simón-Sánchez et al., 2009; Lin and Farrer, 2014). To date, advances in genomics have identified 28 genes associated with PD (Lin and Farrer, 2014).

Several pathways have been linked to the pathogenesis of PD including mitochondrial dysfunction, endoplasmic reticulum stress, autophagy, inflammation and impaired insulin signaling (Mercado et al., 2013; Nolan et al., 2013; Santiago and Potashkin, 2013b; Lin and Farrer, 2014). Despite this progress, the precise disease-causing mechanisms of PD are not fully understood. Complementation of genomic and transcriptomic studies with system biology approaches have provided insights into some novel mechanisms of disease. For instance, differential co-expression network analysis (DCA) performed on transcriptomic data from PD substantia nigra at autopsy, uncovered a transcript isoform of *SNCA* with an extended 3′ untranslated region, termed aSynL, which influenced SNCA accumulation (Rhinn et al., 2012). Interestingly, the pattern of expression of the long aSynL isoform relative to the short isoforms was also observed in unaffected individuals harboring a PD risk variant in the *SNCA* locus (Rhinn et al., 2012).

Understanding the molecular events associated with the progression of PD could help delineate a timeline for effective therapeutic intervention. Gene co-expression network analysis showed differences in gene modules between PD and controls for different anatomic brain regions (Corradini et al., 2014). In PD, hub modules in the motor vagal nucleus, locus coeruleus, and substantia nigra were enriched in pathways related to stress response and neuron survival/degeneration mechanisms whereas in control samples gene modules were associated with neuroprotection and aging homeostasis. Interestingly, one of the main hubs in the substantia nigra of control samples was *SIRT1*, which has been widely implicated in neuroprotection in several neurodegenerative diseases (Donmez and Outeiro, 2013; Herskovits and Guarente, 2013). Analysis of PPI networks representing autophagy and mitochondrial dysfunction pathways identified key protein targets including p62, GABARAP, GBRL1 and GBRL2 that modulated 1-methyl-4-phenylpyridinium (MPP^+^) toxicity (Keane et al., 2015), a widely used toxin to mimic PD in animal and cellular models. Strikingly, overexpression of these proteins combined, but not each one alone, provided rescue of MPP^+^ toxicity. This result further strengthens the notion that targeting a cluster of genes rather than a single gene may be the route to an effective treatment.

Integrative system biology approaches incorporating network analyses have been valuable in identifying potential therapeutic targets in PD. For instance, construction of networks integrating genetic information from Gene expression Omnibus (GEO), the Parkinson’s disease database (ParkDB) and the Comparative Toxicogenomics Database (CTD), identified alvespimycin (17-DMAG) as a candidate neuroprotective agent for PD (Gao et al., 2014). Experimental validation showed that 17-DMAG attenuated rotenone-induced toxicity *in vitro*. Another approach combined human brain and blood transcriptomic data and identified RGS2 as a key regulator of LRRK2 function (Dusonchet et al., 2014), one of the most common genetic risk factor of PD. Of note, RGS2 protected against neuronal toxicity in a *Caenorhabditis elegans* model expressing wild type *LRRK2*. Combination of -omics data from different tissues, for example brain and blood, may be advantageous to understand neurodegeneration in light of the recent finding that demonstrated that cell types outside the brain contain genetic risk factors associated with PD (Coetzee et al., 2016) and thus may help uncover new putative therapeutic targets.

## Network Analysis in Huntington’S Disease (HD)

Huntington’s disease (HD) is one of the most common dominantly inherited neurodegenerative disorders. The symptoms include some motor symptoms, such as chorea and dystonia, as well as non-motor symptoms, including psychological changes, and cognitive decline leading to dementia (Ross et al., 2014). These symptoms are correlated with a selective degeneration of the striatal and cortical neurons (Ehrlich, 2012). Currently, there are no therapies to prevent the onset or slow the progression of HD.

This progressive and fatal disease is caused by abnormal extension of the CAG repeat coding for a polyglutamine (polyQ) tail in the huntingtin gene (*HTT*, MacDonald et al., 1993). Unaffected individuals have fewer than 36 repeats, whereas affected patients can have as many as 250 CAG repeats. It has been shown that the length of the polyQ extension is inversely proportional to the age of the disease onset (Orr and Zoghbi, 2007). Vesicle and mitochondrial transport, transcription regulation, neurogenesis and energy metabolism are among the cellular functions of the normal HTT protein (Borrell-Pagès et al., 2006). Both lost of function of the normal protein and gain of toxic properties of mutant HTT leads to HD pathology. In fact, it has been shown that whereas the normal HTT protein is neuroprotective, the mutant HTT is neurotoxic. Despite this progress, the molecular mechanisms involved in the complex phenotype of the disease are still largely unknown.

In order to understand the role of HTT in HD pathology, (Langfelder et al., 2016) expressed HTT with different CAG length in a mice model. They demonstrated that the length of the CAG repeats modified the transcriptome of the striatum, and to a lesser extent, the cortex. WGCNA allowed the identification of 13 striatal and five cortical gene coexpression modules that were strongly associated with *Htt* CAG length. Interestingly, cadherin and protocadherin (*Pcdh*) genes expression were dysregulated in four of the modules, indicating that regulatory factors of these genes, such as *Rest, Ctcf* and *Rad21*, could be involved in HTT toxicity in mice (Langfelder et al., 2016).

Similarly, WGCNA was performed on transcriptomic HD post mortem tissues including the frontal cortex, cerebellum and caudate nucleus regions. The authors found that genes involved in metalloprotein, stress response and angiogenesis were positively regulated in all the networks whereas genes involved in mitochondrion, glycolysis, intracellular protein transport, proteasome and synaptic vesicle were downregulated (Neueder and Bates, 2014). Analysis of the human transcriptome from HD patients compared to healthy samples confirmed that protein modification, vesicles transport, cell signaling and synaptic transmission are important pathways involved in HD (Mina et al., 2016). Interestingly, these modules were also found in a blood transcriptomic study (Mina et al., 2016). Despite the fact that dysregulation of similar pathways was observed in the blood and brain, there was no overlap in any of the individual genes common between the two tissues (Mina et al., 2016).

A system-based approach performed on human transcriptomic datasets from post mortem human cerebellum, frontal cortex and caudate nucleus from HD patients and controls showed that an astrocyte module is the network whose connectivity and expression is most altered in HD (Scarpa et al., 2016). This astrocyte module was located downstream of TGFβ -FOXO3 signaling. In this regard, the TGFβ pathway was upregulated in neural stem cell differentiated from HD patient induced pluripotent stem cells (iPSC; Ring et al., 2015). Analysis of corrected iPS cells expressing shorter polyQ tails showed a downregulation of TGFβ pathway target genes, including cyclin-dependent kinase inhibitor 2B (*CDKN2B*), inhibitor of DNA binding 2 (*ID2*), inhibitor of DNA binding 4 (*ID4*), paired-like homeodomain transcription factor 2 (*PITX2*), thrombospondin 1 (*THBS1*), and left-right determination factor 2 (*LEFTY2*, An et al., 2012). In addition, valproic acid and lithium, both affecting TGFβ signaling, have been shown to improve mood in HD patients (Grove et al., 2000; Liang et al., 2008; Watanabe et al., 2011; Scheuing et al., 2014; Raja et al., 2015).

HD non-motor symptoms such as stress-related psychiatric and sleep disturbances often precede the onset of motor symptoms (Duff et al., 2007) and system-based approaches have proposed that sleep and stress traits emerge from shared genetic and transcriptional networks (Jiang et al., 2015). Interestingly, the astrocyte network expression described by Scarpa et al also correlated with stress and sleep phenotype in a chronically stressed mouse model (Scarpa et al., 2016). Collectively, these results suggest that targeting components of the TGFβ signaling pathway may provide novel therapeutics for HD.

## Network Approaches to Understand the Connection Among Neurodegenerative Diseases

Widespread protein misfolding and aggregation is a hallmark of neurodegenerative diseases. Despite the fact that neurodegerative diseases are defined by a set of characteristic pathological and clinical features, there is some overlap in pathology, genetic risk factors, and mechanisms of disease. For example, accumulation of SNCA and Lewy body pathology, central in the pathogenesis of PD, are present in the brains of human AD and implicated in aberrant synapse formation (Hamilton, 2000; Kim et al., 2004). Several studies have identified Single nucleotide polymorphisms (SNPs) in the *MAPT* locus associated with PD and AD thus suggesting that a common genetic factor may put an individual at risk for both diseases (Desikan et al., 2015). In addition to *MAPT*, other genetic variants including *PON1, GSTO*, and *NEDD9* have been associated with the risk of PD and AD thus strengthening the genetic overlap between both diseases (Xie et al., 2014). Not surprisingly, shared mechanisms related to oxidative stress, neuroinflammation, impaired insulin signaling, mitochondrial dysfunction, iron dyshomeostasis and nicotinic receptors have been implicated in the pathogenesis of AD and PD (Xie et al., 2014). Therefore, a system-level understanding of the disease-disease connections could accelerate the discovery of novel treatments for both neurodegenerative diseases.

A systems-based approach combining expression quantitative trait loci (eQTL) studies from cerebellum and frontal cortex of AD patients, GWAS from AD and PD and PPI networks indicated that some PD variants (cisSNPs, cis-acting SNPs) were associated with the expression of *CRHR1*, LRRC37A4 and *MAPT* located at 17q21 and suggestive of AD risk (Liu et al., 2015). Similarly, shortest path analysis on a network constructed from literature mining identified known genes that already have an association with AD and PD and seven previously unknown genes including *ROS1, FMN1, ATP8A2, SNORD12C, ERVK10, PRS* and *C7ORF49* that may link both diseases (Kim et al., 2016). Besides finding shared genetic associations, network analysis employing the computation of a similarity matrix identified gene clusters related to DNA repair, RNA metabolism, and glucose metabolism shared in AD and PD (Calderone et al., 2016). Importantly, these pathways were not detected using the conventional gene ontology (GO) analysis thus highlighting the power of networks to uncover novel pathways.

In addition to the studies focused on AD and PD, recent network-based approaches have been applied to understand the molecular networks shared among other neurodegenerative diseases. One study focused on the dorsolateral prefrontal cortex (DLPFC) which is commonly affected in both AD and HD to construct coexpression networks using genome wide expression data from 600 postmorterm DLPFC tissues from AD, HD, and non-dementia controls. Differential coexpression analysis revealed a subnetwork of 242 genes enriched in pathways related to neuron differentiation, apoptosis, gap junction trafficking, and cellular metabolic processes (Narayanan et al., 2014). Interestingly, the 242 gene subnetwork overlapped with genes downregulated in postmortem brains of major depressive disorder, a condition that is associated with other neurodegenerative disorders including PD (Aarsland et al., 2012). Further inspection of this subnetwork identified a gained/lost gene coexpression patterns associated with chromatin organization and neural differentiation.

## Network-Based Approaches to Understand Aging-Associated Neurodegeneration

Aging is one of the most common risk factors associated with neurodegeneration. With an average age of onset of 60 for the most common neurodegenerative diseases, the risk of developing PD or AD significantly increases with age. Dopamine synthesis, a crucial neurotransmitter that becomes depleted in the brain of PD, declines with age (Ota et al., 2006) and amyloid deposits, characteristic pathology in AD, are found in the aging brain of non-demented individuals (Pike et al., 2007). Beyond the overlap in pathological features, aging and neurodegenerative disorders share several dysregulated pathways. A system-based approach that identifies molecular networks shared between aging and neurodegeneration should reveal shared mechanisms, some of which may be targets for slowing disease progression. Discovering unique dysregulataed pathways that are not aging-associated could pinpoint potential therapeutics targets unique for a particular neurodegenerative disease.

Several studies have employed system biology tools to better understand age-related neurodegeneration. For example, a comparative pathway and network analysis of the brain transcriptome revealed shared networks and pathways between aging and PD including inflammation, mitochondrial dysfunction and metal ion homeostasis (Glaab and Schneider, 2015). Interestingly, the expression of the most significant shared gene, *NR4A2*, gradually declined with aging and PD. They found that this aging-associated gene expression changes in *NR4A2* might increase the risk of PD by mechanisms similar to gene mutations linked to PD (Glaab and Schneider, 2015).

Proteostasis functional decline is common in aging and neurodegenerative diseases. In fact, several studies have proposed a mechanistic link between aging and loss of protein homeostasis leading to protein aggregation and toxicity. In this context, chaperones play a pivotal role in protein assembly and folding and its dysregulation may lead to protein aggregation and proteotoxicity. A recent study identified a chaperone subnetwork that exhibited concordant repression and induction expression patterns in brain tissues from human aging, AD, HD and PD patients. Subsequent investigation led to the discovery of a subnetwork comprising HSC70, HSP90, the CCT/TRiC complex and HSP40 and TPR-domain related co-chaperones with aberrant expression that were required to prevent Aβ and polyQ-associated proteotoxicity in *C. elegans* (Brehme et al., 2014). This shared chaperome subnetwork in aging and neurodegeneration, which is critical to maintain protein homeostasis, provides new targets for therapeutic intervention in neurodegenerative diseases. Similarly, a recent meta-analysis of about 1600 microarrays from brain tissue of AD patients revealed a set of downregulated genes corresponding to metastable proteins prone to aggregation (Ciryam et al., 2016). Thus, targeting components of the proteome homeostasis network may enable novel therapeutic opportunities for neurodegenerative diseases.

## Epigenetics, Aging and Neurodegenerative Disorders

Gene expression is temporally and spatially regulated by DNA methylation or histone modifications. These epigenetic changes could influence a global gene expression or target some specific genes. A role for epigenetic changes in gene expression has been proposed in aging and neurodegenerative disorders. Interestingly, many studies have reported a genome-wide tendency to DNA hypomethylation with age in different organs including the brain in aging animal models (Wilson et al., 1987; Brunet and Berger, 2014). These changes are proposed to play a role in the progression of aging (Benayoun et al., 2015; Zampieri et al., 2015). Interestingly, Humphries et al. (2015) has shown that hypomethylation was observed in a myelination network dysregulated in AD.

DNA methylation has been proposed as a biomarker for aging in cells, tissues and organs (Horvath, 2013). An acceleration of the epigenetic clock has been proposed in different neurodegenerative disorders. In this context, epigenetic age acceleration correlated with AD neuropathological markers such as neuritic plaques and amyloid load (Levine et al., 2015). In addition, an association between epigenetic age acceleration with episodic memory, working memory and cognitive decline was observed among individuals with AD (Levine et al., 2015). Histones modifications, such as acetylation and methylation, have been observed in AD models and patients (for review see Fischer, 2014). Interestingly, the epigenetic clock is also accelerated in brain regions from HD patients (Horvath et al., 2016).

Epigenetic modification is also proposed to contribute to neurodegeneration in PD. A genome wide DNA methylation and transcriptomic study in iPSC-derived dopaminergic neurons from *LRRK2*-associated PD patients identified common DNA methylation changes in *LRRK2* and sporadic PD (Fernández-Santiago et al., 2015). DNA methylation changes in PD dopaminergic neurons correlated with the downregulation of RNA and protein expression of a network of transcription factors *FOXA1, NR3C1, HNF4A* and *FOSL2*, which have been implicated in PD. For instance, FOXA1 is a key determinant in the molecular and physiological properties of dopaminergic neurons (Pristerè et al., 2015) and *HNF4A* expression in blood has correlated with disease progression in PD (Santiago and Potashkin, 2015).

Several computational tools have been developed to facilitate the integration of epigenetic data in networks. For example, EpiRegNet is a publicly available web server that allows the construction of epigenetic regulatory networks from human transcriptomic data (Wang et al., 2011). Another model, the Artificial Epigenetic Regulatory Network (AERN) incorporated DNA methylation and chromatin modification in addition to genetic factors for the analysis of epigenetic networks (Turner et al., 2013). More recently, another computational model, the Biological Expression Language (BEL)^3^, enabled the analysis of functional consequences of epigenetic modifications in the context of disease mechanisms (Khanam Irin et al., 2015). Because BEL integrates literature-derived cause and effect relationships into networks, researchers can formulate novel hypotheses of disease mechanisms. Notably, BEL network modeling has been used to integrate epigenetic and genetic factors in a functional context in PD. Using this approach, *SNCA, MAPT, DNMT1, CYP2E1, OLFR151, PRKAR2A* and* SEPW1*, were found to be hypomethylated in PD and suggested to cause overexpression of genes that disrupt normal biological functions. Further, two SNPs, rs3756063 and rs7684318, were associated with hypomethylation of SNCA in PD patients (Khanam Irin et al., 2015). Collectively, these models demonstrate that the integration of epigenetic factors into networks can uncover novel mechanisms of disease.

## Challenges and Future Directions in Network Medicine Applications Towards Personalized Treatment

The field of network medicine has undoubtedly accelerated the understanding of the molecular mechanisms leading to neurodegeneration. The most significant brain network-based studies of the most common neurodegenerative diseases are summarized in Table 2. While network-based methods provide an unbiased approach to decode complex diseases and generate novel hypothesis, experimental validation is essential for network findings to be translated into useful diagnostics and therapeutic applications. In this regard, a growing number of studies have successfully identified blood-based biomarkers with potential clinical applicability. For instance, network analysis identified *SOD2, APP, HNF4A, PTBP1* and *NAMPT* as useful to distinguish PD patients from HC in blood samples obtained from two independent cohorts (Santiago and Potashkin, 2013a, 2015; Santiago et al., 2014, 2016). Among these biomarkers, *HNF4A* and *PTBP1*, showed a dynamic expression pattern in longitudinal samples thus showing potential to track the clinical course of PD patients. Likewise, network analysis identified *PTPN1* as a useful blood biomarker to distinguish PD from progressive supranuclear palsy, an atypical parkinsonian disorder commonly misdiagnosed as PD (Santiago and Potashkin, 2014b). Despite the success in PD studies, experimental validation of network-based findings in AD and HTT studies in clinically relevant studies is mostly lacking. For example, a systems medicine approach identified TYROBP as a promising target for therapeutic intervention in AD but to the best of our knowledge there are no follow-up studies (Zhang et al., 2013). Similarly, the involvement of *RORA* (Acquaah-Mensah et al., 2015) and other potential targets in AD are yet to be validated.

Besides experimental validation, another aspect for consideration is the cell-type and tissue specific analysis. This is important since the analysis of gene expression studies from whole brain sections might lead to misleading results that are not relevant to the specific cell type affected in the disorder. To circumvent this problem, recent studies have successfully employed high-throughput technologies that enable a single-cell resolution. A notable example studied the changes in astrocyte and microglia reactivity in AD. They observed that genes within the immune response pathway were more pronounced in astrocytes than in microglia thus demonstrating that cell-type specific characterization of the molecular changes may be more informative (Orre et al., 2014). More details about limitations in system-biology approaches in the context of neurodegenerative diseases have been well described recently (De Strooper and Karran, 2016).

**Figure 3:**
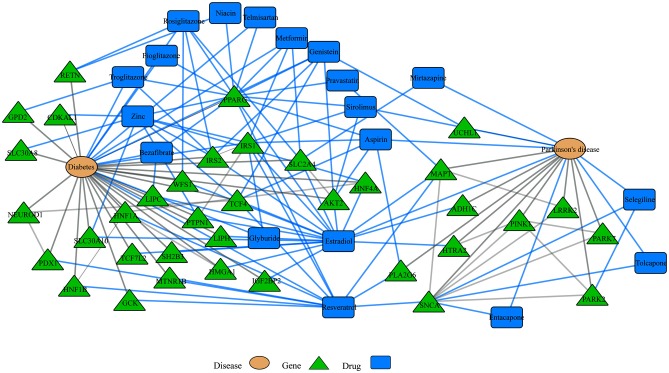
Disease-drugs networks. Interaction among different diseases, drugs and genes can be represented in a multi-level network model. For example, network-based approaches have been used to understand shared dysregulated pathways in Parkinson’s disease (PD) and diabetes. For instance, some drugs to treat diabetes patients have shown neuroprotective effects in PD and the observed neuroprotection may be mediated through their interaction with the peroxisome proliferator-activated receptor gamma (PPARG). Blue and gray lines represent drug interactions and disease interactions, respectively. This network was retrieved by iCTNet application in Cytoscape v3.1.1. using genetic associations from genome wide association studies (GWAS) and drug interactions from the Comparative Toxicogenomics Database (CTD) as of September 2016.

Another emerging area of research in the network biology field is the study of disease comorbidities. Several conditions including, diabetes, cancer, major depressive disorder and cardiovascular disease, for example, have been associated with neurodegenerative diseases. For example, insulin resistance and diabetes have been linked to AD and PD and drugs to treat diabetic patients have shown promising results in both disorders (Santiago and Potashkin, 2013b). In addition, analyses of shared networks between PD and diabetes have elucidated potential blood biomarkers for PD (Santiago and Potashkin, 2013a, 2014c; Santiago et al., 2014). More recently, an integrative transcriptomic meta-analysis of PD and major depression identified *NAMPT* as a potential blood biomarker for *de novo* PD patients (Santiago et al., 2016). Furthermore, treatment with an enzymatic product of NAMPT elicited neuroprotective effects via activation of SIRT1 in an *in vitro* model of PD (Zou et al., 2016). Therefore, understanding the molecular networks shared between comorbid diseases could reveal novel diagnostics and therapeutic targets. Network analysis of gene-drug interactions in PD and diabetes demonstrates that some drugs may be beneficial for treating both diseases (Figure 3). In this context, treatment with commonly prescribed drugs to treat diabetes including rosiglitazone, metformin, pioglitazone and exenatide have shown neuroprotective effects in PD models (Santiago and Potashkin, 2013b, 2014c; Aviles-Olmos et al., 2014; Carta and Simuni, 2015). In particular, treatment with exenatide improved motor and cognitive function in PD patients (Aviles-Olmos et al., 2013, 2014). Treatment with pioglitazone, however, did not result in disease-modifying benefits in PD patients (NINDS Exploratory Trials in Parkinson Disease (NET-PD) FS-ZONE Investigators, 2015; Simon et al., 2015). Nonetheless, it has been noted that a longer exposure to pioglitazone may have been required to observe an improvement in PD patients (Brundin and Wyse, 2015). As shown in Figure 3, interaction of these drugs with the peroxisome proliferator-activated receptor gamma (PPARG) may provide a mechanistic explanation to their neuroprotective effect. Some of these drugs are currently in clinical trials to determine if they are neuroprotective.

Nutrition is also recognized as an important component in the development and treatment of neurodegenerative diseases (Seidl et al., 2014). Given the promise of neuroprotective agents, the field of nutrigenomics is gaining interest among neuroscientists that are seeking to understand the complex nutrient-genetic interactions underlying neurodegeneration and neuroprotection. A recent example conducted a transcriptomic and epigenomic sequencing of the hypothalamus and hippocampus from a rodent model exposed to fructose consumption, which has been shown to contribute to the metabolic syndrome (Meng et al., 2016). Gene network analysis identified *Bgn* and *Fmod* as key genes involved in the observed metabolic alterations induced by fructose in mice. Strikingly, administration of docosahexaeonic acid (DHA) reversed the gene network changes elicited by fructose (Meng et al., 2016). This study provides evidence that integration of nutrigenomics coupled with network analysis can facilitate the identification of neuroprotective agents. Likewise, resveratrol, an antioxidant present in red wine, may also provide neuroprotection in PD patients and thus, could be tested in clinical trials (Figure 3).

In addition to a nutrient-rich diet, both physical exercise and cognitive training promote healthy aging (Kraft, 2012; Bamidis et al., 2014). It has been proposed that a combination of both together may be best to prevent cognitive decline and pathological aging (Kraft, 2012; Bamidis et al., 2014). In this regard, network analysis could be a useful tool to characterize the effects of physical exercise and cognitive training in the aging brain. Future studies directed at identifying gene expression changes associated with these lifestyle changes would be advantageous. Collectively, a multidimensional network approach that includes information about symptoms, drug treatments, comorbidities, nutrigenomics, physical exercise and cognitive training will be valuable to accelerate personalized treatment.

## Author Contributions

JAS and VB wrote the first draft of the manuscript. JAS, VB and JAP edited and reviewed the final draft of the manuscript.

## Conflict of Interest Statement

The authors declare that the research was conducted in the absence of any commercial or financial relationships that could be construed as a potential conflict of interest.

## Footnotes

^1^http://www.alzheimers.net/resources/alzheimers-statistics/

^2^http://www.alsgene.org/

^3^http://www.openbel.org/
